# Men report good mental health 20 to 23 years after in vitro fertilisation treatment

**DOI:** 10.1186/s12889-015-2398-6

**Published:** 2015-11-25

**Authors:** Gunilla Sydsjö, Josefin Vikström, Marie Bladh, Barbara Jablonowska, Agneta Skoog Svanberg

**Affiliations:** Division of Obstetrics and Gynaecology, Department of Clinical and Experimental Medicine Faculty of Health Sciences, Linköping University, SE-581 85 Linköping, Sweden; Department of Women’s and Children’s Health, Uppsala University, S-751 85 Uppsala, Sweden

**Keywords:** Infertility, *In vitro* fertilisation, Mental health, Men, Long term

## Abstract

**Background:**

Infertility and infertility treatment are known to have negative short-term psychological consequences for men and women, with more long-term consequences for women. The long-term wellbeing and mental health of men who have experienced *in vitro* fertilisation (IVF) treatment has not been extensively described in the literature. Therefore, the aim of this study was to analyse the mental health of men 20 to 23 years after IVF treatment.

**Method:**

The Symptom Checklist 90 tool was used to assess the self-perceived mental health of men who were part of a couple that underwent IVF treatment at Linköping University Hospital, Sweden, 20 to 23 years earlier. We enrolled 292 out of the 490 men who took part in the hospital’s IVF programme from 1986 to 1989 and compared them to an aged-matched control group. In addition, the men who had remained childless were compared to those who had fathered biological children and those who had adopted children.

**Results:**

The overall mental health of the men who had received IVF was good. We found that 54 % of the men had fathered their own biological children, 21 % were childless and the remainder were part of a couple that had gone on to adopt. The childless men displayed more mental health problems than the other men in the study, as did men who were unemployed, single or divorced.

**Conclusion:**

This study carried out 20 to 23 years after IVF treatment showed that the majority of the men who took part were in good mental health. Those who remained childless faced an increased risk of negative psychological symptoms and men who were single showed more symptoms of depression and anxiety disorders.

## Background

The long-term mental health and wellbeing of men who have experienced infertility and infertility treatment is under-reported in the scientific literature. Until now, the emphasis has been on the short-term consequences of infertility for men and women or the long-term consequences for women. For the most part, the studies show a different pattern for men and women regarding their mental health and emotional reactions, with higher short-term incidences of depression and anxiety symptoms in women [1, 2]. However, Fisher and Hammarberg carried out a review of 73 studies on men’s reactions to their infertility diagnoses and treatment for infertility. They concluded that men who had been diagnosed with infertility wanted to be parents and had the same reactions as women if infertility treatment was not successful [3]. Also, a Finnish population-based study by Klemitti et al., of 2291 men and women aged 30 to 44 years, found that infertile and childless men experienced a significantly poorer quality of life than men without infertility problems or diagnoses [4].

Men, as well as women, can be affected by their infertility in different ways. They may have a partner who is infertile, be part of a couple where the infertility is unexplained or be the one who has been diagnosed with infertility, such as with oligospermia or azoospermia. A study of men with non-obstructive azoospermia and their partners, showed that the men were negatively affected by their diagnosis, with regard to their sexual function as well as their physical and psychological well-being [5]. One of the few previous studies on the long-term psychological effects of *in vitro* fertilisation (IVF) on men found that those who had experienced unsuccessful IVF treatment were more depressed and had a lower level of general wellbeing and quality of life than men who became fathers after treatment [6]. Also, infertility was still a central issue for the couples five years after they stopped their unsuccessful treatment [7].

The aim of this study was to compare the mental health of men who had had experienced IVF 20 to 23 years earlier to the mental health of men of the same age in the general population. The second aim was to study the possible difference in mental health between two groups: those who became fathers after treatment or through adoption and those who remained childless.

## Method

### Subjects

We sent an introductory letter and study invitation to all of the 490 men who, together with their partner, had been through at least one IVF cycle at the Center of Reproductive Medicine at the University Hospital in Linkoping between 1986 and 1989. Of these, 11 had died and a further 187 either declined to take part, did not receive the letter or did not answer the questionnaire. This left 292 participants and a participation rate of 66.5 %. We divided the men into three groups: those who had fathered a child after treatment, those who said they had remained childless and those who had adopted one or more children. As a reference group, we used 123 men from a population-based study of men over 40-years-of-age, which had validated use of the same tool we used in this study, the Symptom Checklist 90 tool (SCL-90) [8].

Between 1986 and 1989, couples accepted for IVF were given three or more publically funded treatment cycles. The couples could have adopted or biological children before receiving the treatment. The upper age limit at the time of treatment was 38 years for the women, with no age limit for the men.

A study-specific questionnaire was used to collect information on; age, occupation, employment status, marital status, number of biological and adopted children, number of miscarriages the men’s partner had had and the number of IVF treatment cycles they had received. The men’s mental health was measured with the SCL-90, which is a commonly used multi-dimensional psychological status symptom inventory consisting of 90 items [9]. The SCL-90 provides an objective method for symptom assessment, with the individuals rating their own psychopathological problems and symptoms, such as depression and anxiety. The scale is a five-point Likert scale ranging from “not at all” (0) to “extremely” (4). The SCL-90 assesses nine primary dimensions: somatisation, obsessive compulsive, interpersonal sensitivity, depression, anxiety, hostility, phobic anxiety, paranoid ideation and psychoticism. There is also a global index of distress that can be used as a summary dimension. In order to provide a reference measurement for the SCL-90, we used a group of Swedish men in the same age groups as the participating men to compare the scores [8]. There was no information about the men in the reference group regarding their social status or if they had children or had experienced childlessness nor was any information regarding infertility treatment available.

#### Statistics

Socio-demographic background data were summarized with absolute number and percentages, while the nine different subscales of SCL-90 (somatisation, obsessive compulsive, interpersonal sensitivity, depression, anxiety, hostility, phobic anxiety, paranoid ideation and psychoticism) and three summary scales (global severity index, positive symptom distress index, and positive symptom total) were summarized with mean/SD as well as median/minimum-maximum values.

A multivariate ANOVA (MANOVA) was performed to analyse the combined effect of the independent variables (participants’ employment, marital status, age-at-follow-up, whether they were with the same partner as when they had the IVF and whether or not they had children) on all 12 scales of SCL-90. Subsequent univariate ANOVAs were performed on each of the subscales to further elucidate the importance of socio-demographic factors on SCL-90 (where the MANOVA had indicated statistical significant effects).

The Student’s *t*-test was used to carry out separate comparisons of the mean scores of the nine subscales between the men who had received IVF and the men in the reference group but also between those who had become fathers and those who were still childless. Due to multiple testing a Bonferroni adjusted a two-sided *P*-value of <0.002 were considered statistically significant. All statistical analyses were performed using IBM SPSS version 22 (IBM Corporation, Armonk, NY).

#### Ethics

The study was approved by the Regional Ethical Review Board in Linköping Sweden.

## Results and discussion

A total of 292 men who had undergone IVF treatment 20 to 23 years earlier in 1986 to 1989 answered the SCL-90 questionnaire. On average, each couple had experienced 2.5 (range = 1–10) IVF cycles and 108 of the men had partners who had previously experienced between one and seven miscarriages (mean = 2.0) (data not shown).

The men’s demographic data are presented in Table 1. The majority of the men were employed, married and lived with the same partner as when they had the IVF treatment. More than half (54 %) had biological children and 21 % did not have any children. The remainder were part of a couple who had adopted children. Summary statistics of the 12 different scales derived from the SCL-90 questionnaire are presented in Table 2. In general, most men assessed their mental health as good.

**Table 1:** Demographic variables of the 292 men studied

		*n*	%
Employment status	Employed	248	84.9
	Unemployed	27	9.2
	Missing	17	5.9
Marital status	Married/cohabiting	257	88.0
	Separated/divorced/other	35	12.0
Same partner	Yes	219	75.0
	No	42	14.4
	Missing	31	10.6
Children	No children	62	21.1
	Biological children	159	54.5
	Adoptive children	51	17.5
	Biological and adoptive children	20	6.8
Age at follow-up	<45 years old	76	26.0
	> = 45 years old	216	74.0

**Table 2:** Descriptive statistics for the different dimensions of SCL-90

	Mean (SD)	Median	Min-Max
Somatization	1.35 (2.68)	0.00	0.00–21.00
Interpersonal Sensitivity	2.75 (3.94)	1.00	0.00–22.00
Depression	5.48 (7.25)	3.00	0.00–48.00
Anxiety	3.54 (4.73)	2.00	0.00–29.00
Hostility	1.53 (1.98)	1.00	0.00–13.00
Phobic anxiety	0.55 (1.68)	0.00	0.00–15.00
Paranoid Ideation	1.49 (2.64)	0.00	0.00–16.00
Psychoticism	1.35 (2.68)	0.00	0.00–21.00
Obsessive-Compulsive	4.63 (5.52)	3.00	0.00–34.00
Global Severity Index	29.52 (33.86)	17.50	0.00–202.00
Positive Symptom Distress Index	0.33 (0.38)	0.20	0.00–2.27
Positive Symptom Total	19.75 (16.69)	15.00	1.00–82.00

The MANOVA analysis, presented in Table 3, indicated a relationship between employment status and marital status on all 12 subscales of SCL-90. However, no differences in self-perceived mental health status were found between those without children and those with biological or adopted children nor did age affect their self-perceived mental status, but the men who were divorced, single or unemployed displayed more mental health problems. Men who were unemployed were more likely to exhibit symptoms of somatization, hostility, psychoticism, obsessive-compulsive they also had a higher Positive Symptom Distress Index, and Global Severity Index (Table 4).

**Table 3:** Multivariate tests of the combined effect of all independent variables on the combined outcome of all 9 subscales of SCL

Effect^a^		Value	F	Hypothesis df	Error df	Sig.	Partial Eta Squared
Employment	Pillai’s Trace	.102	2.265	11.000	220.000	0.012	0.082
Marital status	Pillai’s Trace	.111	2.488	11.000	220.000	0.006	0.095
Same partner	Pillai’s Trace	.065	1.400	11.000	220.000	0.174	0.025
Children	Pillai’s Trace	.117	0.818	33.000	666.000	0.756	0.025
Age at follow-up	Pillai’s Trace	.064	1.358	11.000	220.000	0.195	0.027

^a^Using Pillai’s trace estimates

**Table 4:** Tests of between-subject effects, from 12 separate univariate ANOVAS, model statistics, estimated differences and corresponding statistical significance

Independent variable	Subscale	Mean Square	F	Unemployed vs. employed Difference	Divorced/single vs. Married/cohabiting Difference	*p*-value
Employment	Somatization	53.897	9.605	1.733		0.002
	Interpersonal Sensitivity	40.183	3.021	1.531		0.084
	Depression	318.873	8.022	4.312		0.005
	Anxiety	133.671	6.945	2.792		0.009
	Hostility	47.046	11.755	1.656		0.001
	Phobic anxiety	3.450	1.426	0.449		0.234
	Paranoid Ideation	35.048	5.673	1.430		0.018
	Psychoticism	53.897	9.605	1.773		0.002
	Obsessive-Compulsive	307.099	11.529	4.232		0.001
	Positive Symptom Total	1393.798	5.937	9.015		0.016
	Positive Symptom Distress Index	1.292	11.223	0.274		0.001
	Global Severity Index	10470.795	11.279	24.709		0.001
Marital status	Somatization	77.922	13.886		3.850	<0.001
	Interpersonal Sensitivity	120.726	9.076		4.793	0.003
	Depression	324.505	8.164		7.857	0.005
	Anxiety	76.446	3.972		3.814	0.047
	Hostility	4.100	1.024		0.883	0.313
	Phobic anxiety	18.730	7.740		1.888	0.006
	Paranoid Ideation	22.101	3.578		2.051	0.060
	Psychoticism	77.922	13.886		3.850	<0.001
	Obsessive-Compulsive	187.859	7.053		5.978	0.008
	Positive Symptom Total	1357.950	5.785		16.074	0.017
	Positive Symptom Distress Index	0.854	7.422		0.403	0.007
	Global Severity Index	6751.357	7.273		35.840	0.008

In addition, being divorced or single had a negative impact on somatization and. psychoticism. When we compared the IVF men with men from the general population, few statistically significant differences were detected. However, IVF men had an increased mean score on the depression subscale, as well as on the Global Severity Index and as well on the Positive Symptom Distress Index (Table 5), though the latter do not quite reach statistical significance. Overall, there appeared to be a tendency towards men without children having a more vulnerable mental status than the control group and than the IVF men who became fathers (Table 5). The interpretation of this results is difficult since we do not know these men’s health status over the years nor do we know if being childless interfere with mental health or if mental interfere the chance of having a family and experience parenthood with biological or adopted children.

**Table 5:** The study group of 292 men compared to the reference group of 123 age-matched men

	IVF men *n* = 292		IVF men without children *n* = 62		IVF men with children *n* = 230		Reference group from a Swedish age >40 years *n* = 123			
	Mean	Std. deviation	Mean	Std. deviation	Mean	Std. deviation	Mean	Std. deviation	*p*-value*	*p*-value w/ or w/o children
Somatisation	0.33	0.59	0.40	0.66	0.31	0.56	0.30	0.34	0.369	0.315
Obsessive-Compulsive	0.39	0.62	0.52	0.67	0.36	0.61	0.38	0.41	0.784	0.097
Interpersonal Sensitivity	0.23	0.52	0.34	0.57	0.20	0.50	0.26	0.34	0.326	0.072
Depression	0.35	0.63	0.55	0.80	0.29	0.57	0.28	0.35	0.048	0.004
Anxiety	0.31	0.57	0.44	0.67	0.28	0.54	0.25	0.29	0.056	0.059
Hostility	0.21	0.42	0.19	0.40	0.13	0.38	0.19	0.25	0.404	0.782
Phobic Anxiety	0.06	0.27	0.13	0.38	0.03	0.23	0.07	0.20	0.511	0.015
Paranoid Ideation	0.25	0.53	0.40	0.61	0.21	0.50	0.27	0.35	0.521	0.010
Psycoticism	0.09	0.32	0.14	0.44	0.08	0.28	0.10	0.16	0.569	0.152
Global Severity Index	0.33	0.38	0.42	0.45	0.31	0.37	0.25	0.25	0.000	0.095
Positive Symptom Distress Index	1.34	0.47	0.41	0.44	0.31	0.35	1.25	0.41	0.003	0.089
Positive Symptom Total^a^	19.75	16.69	24.13	18.80	18.56	15.91	18.38	14.22	0.203	0.039

*Comparison of IVF men and reference group

^a^Unstandardized score whereas standardized scores were used for the other subscales

The main findings of this study suggest that men who experienced IVF treatment more than 20 years ago were in good mental health. However, compared to an aged-matched reference group of men, the IVF men showed a slightly increased tendency to exhibit symptoms of depression and the men who were childless displayed more mental illness. Men who were divorced or unemployed were vulnerable and more prone to exhibiting symptoms of anxiety and depression, in terms of scoring higher on somatization, hostility, psychoticism, obsessive-compulsive and the Positive Symptom Distress Index as well as the Global Severity Index. Those who had remained childless also reported more symptoms than those who were fathers, though not always reaching statistical significance. Having adopted or biological children might have a protective influence on mental illness in males and females and also on couples’ relationships. The reasons for living alone or being childless might be due to the person’s mental and physical status, but also the effect of being childless [10].

Reactions to infertility and unsuccessful treatment are, of course, individual, but the few studies available on men’s reactions have shown that, in the short-term, men respond to being infertile and childless in the same way that women do, with both physical and psychological negative effects [3]. A 2007 review by Williams et al. concluded that unsuccessful fertility treatment was a risk factor for developing severe depressive symptoms and possibly major depression and that this could have negative long-term medical and social consequences as well as impairing the couple’s relationship. However, no evidence of severe long-term consequences of unsuccessful treatment on men was found in the present study. Also, a previous long-term follow-up study on couples’ relationships 20 years after stopping IVF treatment showed stable relationships and that couples who remained childless showed better communication skills and also better conflict management abilities than couples with children [11].

There are a few previous studies on the health and quality of life of elderly or middle-aged people that have focused on whether childlessness, whether it was through choice or not, had a long lasting impact on their health and wellbeing. A 2011 study by Vikström et al. of people over 85-years-old found no differences in psychological wellbeing between the men and women who were childless and those who were parents [12]. The authors, therefore, concluded that childless elderly individuals found ways to cope with whatever negative effects of childlessness they may have experienced [12].

It could be that the men who had previously experienced infertility had subsequently had time to reflect, build relationships with their partners and cope with their experiences so that their childlessness did not negatively affect their wellbeing. In a longitudinal study from Denmark on relationship in couples coping strategies showed that different and better coping strategies occur for one-third of couples being subjective to a unsuccessful IVF treatment and that emotional support from spouses reinforce this opinion in the couples [13].

The men in this study all received IVF between 1986 and 1989. At that time the indications for IVF treatment were usually female or unexplained factors. Treating male-diagnosed infertility with, for instance, intracytoplasmic sperm injection, was not an option at the time. Also, the men in the couples who underwent treatment all used their own gametes.

One major limitation of this study is that we have no knowledge of the men’s mental state before treatment or during the treatment period. Nor do we have any knowledge about their lives during the many years since the IVF treatment or any idea of the life events or somatic illnesses they may have experienced. We also have reason to suspect that the men who chose not to answer the SCL-90 had more mental health problems and were more affected by their infertility and childlessness. There is also a possibility that their infertility and childlessness was a reason for being single. Participation rates in long-term cohort studies are a problem, with decreasing rates during the last two decades [14]. A 2012 study of dropouts in infertility studies by Troude et al. showed, not surprisingly, that having a child after treatment was the strongest reason why people dropped out of follow-up studies [15]. Therefore, there is a risk that we could have under-estimated the accurate self-perceived mental health in the men from this three-year cohort. It should also be pointed out that the method used to study their mental health, the SCL-90 scale, has limitations as well as strengths.

Men seem to need as much support as women in both the long-term and short-term and it is important for health care professionals to discuss childlessness, infertility and treatment experiences when meeting men in different health care situations.

In the short term it seems that men going through IVF would like more support from the medical professionals who carry out the IVF treatment and they would prefer not to be counselled by social workers [3].

## Conclusion

In conclusion, the results show that most men who had been through IVF in general were in good mental health but that those who remained childless were at risk of negative psychological symptoms. Thus, it might be important in general health care situations to include questions about the family situation and previous experiences of infertility and unsuccessful IVF treatment. Some men might find it a relief to talk about their experiences of childlessness as well as their experiences of infertility, investigations and treatment.
